# Rooting for data: A FAIR research data management and data analysis environment for the PlantMicrobe research collaboration

**DOI:** 10.1371/journal.ppat.1013748

**Published:** 2025-12-08

**Authors:** Frederik Dröst, Ursula Eberhardt, Alexander Wellmann, Halima Saker, Simon Pirkl, Matthias Krinninger, Andreas Klingl, Eric Kemen, Nadia Kamal, Stephan Hachinger, Jens Krüger

**Affiliations:** 1 Chair of Computational Plant Biology, Technical University of Munich (TUM), Munich, Germany; 2 Zentrum für Datenverarbeitung (ZDV), University of Tübingen, Tübingen, Germany; 3 Leibniz Supercomputing Centre (LRZ), Bavarian Academy of Sciences and Humanities, Garching, Germany; 4 Faculty of Biology, Plant Development, Ludwig-Maximilians-Universität München (LMU), Munich, Germany; 5 Center for Plant Molecular Biology (ZMBP), University of Tübingen, Tübingen, Germany; University of Tübingen: Eberhard Karls Universitat Tubingen, GERMANY

Research Data Management (RDM) based on the FAIR principles (Findable, Accessible, Interoperable, and Reusable for both humans and machines [1]) has become a requirement for fundable research [2]. In the DFG Collaborative Research Centre TRR356 “PlantMicrobe”, the cellular and molecular basis of plant-microbe interactions is investigated in model plants and crops. The data generated in the research process will be highly valuable for scientific re-use, e.g., as a reference matrix for other organisms, or because it contains more information than can be extracted by addressing a single research question, for example, in studies investigating the assembly and perturbation of microbial communities *in planta*. The focus of RDM is increasing the reproducibility of data, thus supporting its re-use. Additional impetus for RDM comes from collaborative research. Often, large data volumes need to be stored and made available to diverse groups of users. Different users working in parallel on the same datasets require mechanisms for versioning and data annotation. These factors have led to the establishment of the IT-infrastructure project “VERDA”—a “Virtual Environment for Research Data and Analysis”, which implements best-practice RDM and a tailored compute/data environment for collaboration within the TRR356. Below, we outline the principles behind VERDA [3,4].

## What is the purpose of a Research Data Management Plan (RDMP)?

RDMPs describe the kind and volume of data generated in a project, along with the planned strategy for managing it, usually focusing on the FAIR principles. The principal investigator of a project is responsible for the RDMP, and various tools are available to assist them in creating these plans [5]. Increasingly, RDMPs are required by research agencies before a project is initiated and should be regularly adjusted during the project. Rather than just being a formality, RDMPs are an important tool for planning and ensuring the availability of tailored research data and computing infrastructure during and after the project period.

## How are the researchers being supported regarding RDM?

While RDM processes described in an RDMP may seem logical across the data lifecycle, their practical implementation often presents significant challenges. VERDA addresses these through coordinated efforts of several specialized teams, supporting the diverse needs of TRR356 researchers. The infrastructure and RDM project “VERDA” is organized across all participating institutions with teams that design, implement and manage services like central data storage, Electronic Lab Notebooks, OMERO, and Virtual Machines, while also ensuring data security and access restrictions. Complementing these efforts are dedicated data stewards who work closely with all researchers in the consortium to assess data volumes and types, support data storage, transfer, and archiving and address gaps in the scientists’ RDM knowledge. These efforts directly support integrative projects such as those characterizing host-specific microbiota dynamics across genotypes and environments. This includes general assistance with creating RDMPs, organizing workshops on requested topics, from basic data handling and the use of workflow management systems to metadata standards and ontologies. Additionally, one-on-one support is available for specific use-cases and data constellations.

## What RDM resources are available for plant and microbe research in Germany?

The National Research Data Infrastructure (NFDI) initiative is dedicated to promoting FAIR data principles and developing concepts for reproducibility and data archiving across Germany. NFDI services and resources have become an essential part of RDM solutions for research projects. This initiative is based on 26 subject-specific consortia, collectively working to enhance data management practices. For plant-microbe interaction research, TRR356 is supported by offers and services of several consortia. DataPLANT (NFDI4Plants) offers original resources for plant science, like the Annotated Research Context (ARC) standard, and provides integration into their existing infrastructure offers, creating a basis for mutual benefits and exchange. NFDI4Microbiota contributes metadata standards and templates tailored to microbiome-related datasets, crucial for the documentation of host-specific microbial interactions. Additionally, exchanges with NFDI4BIOIMAGE and their existing documentation support the integration of imaging data via OMERO, including collaborative efforts to enhance compatibility with ARC-based workflows.

## How does the Annotated Research Context (ARC) support FAIR data in plant research?

**Fig 1:**
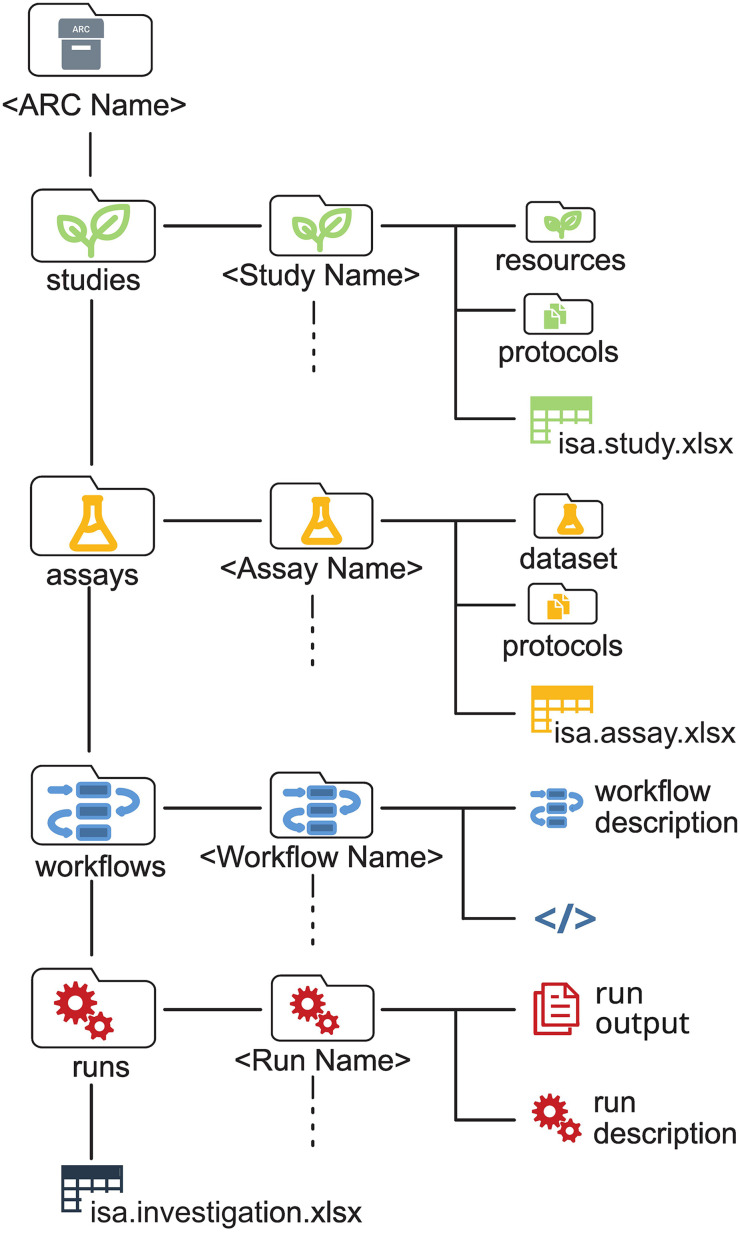
File and folder structure of the ARC (Annotated Research Context). “Studies” include data and metadata of input materials, “assays” of measurement results, in “workflows,” computational workflows are described, and “runs” contain the results of these analyses. Each of these main folders can contain any number of subfolders. Metadata and data are separated, with metadata annotations contained in isa.investigation.xlsx, isa.study.xlsx, and isa.assay.xlsx [9], following the ISA standard and specifically its ISA-tab implementation with spreadsheets. Folder names are fixed, except for those in between angle brackets. The ARC concept is subject to further development. Redrawn and slightly modified from Weil and colleagues [10], copyright by the authors, left-hand panel of their Fig 3, used and licensed under the CC BY 4.0 license (https://creativecommons.org/licenses/by/4.0/).

A core element of FAIR data is the integration of data, metadata, and persistent identifiers (PIDs), including their relationships to support the reproducibility of a study and its assets. This integration happens either through dedicated registries or by combining metadata and data in storage. In the context of plant research, the ARC standard (Fig 1), developed within DataPLANT [6], fulfills this role. An ARC gathers all data related to an investigation and is constructed at the storage level, using a fixed folder structure with specific rules for depositing and annotating data and metadata using ontologies. This folder structure, paired with an elaborate version control system such as Git/GitLab [4], differentiates a typical ARC-based RDM system from, e.g., Fairdom-Seek, an older RDM system built around similar data standards [7].

Following the ISA Abstract Model [8], ARCs consist of folders for data and metadata pertaining to the input of the investigation (studies), wet lab and other analysis results (assays), generalized workflows, scripts, or tool descriptions (workflows), ideally using Common Workflow Language (CWL), and their results (runs). While CWL enhances reproducibility, its use is not mandatory within TRR356, in particular within projects where the complexity of computational workflows is limited. The publication of complete datasets, validated results and any scripts used is prioritized in VERDA. Administrative and scientific metadata are captured in ISA-tab spreadsheets, a tabular format widely used in the life sciences to record experimental design, sample details and assays, ensuring machine-readability and completeness with curated templates. Following the ARC specification rules [9] allows the ARC to be machine-readable, even if CWL is not used. Various tools like ARCitect or ARCmanager [3] assist users with the creation of ARCs.

## What are DataHUBs and how do they facilitate ARC sharing?

ARCs are intended to be shared through DataHUBs [11], built on GitLab infrastructure. Git and GitLab provide robust version control and access management within the ARC ecosystem. During an ongoing study, access permissions can be configured to restrict both data visibility and the ability to manipulate or edit data to specific users. When preparing for publication, a curated version of the ARC can be made publicly accessible to facilitate data reuse. While public access can be granted through GitLab, we recommend publishing an ARC export in a data repository (institutional, domain-specific, or general), to obtain a globally unique PID for the dataset.

## A DataHUB and computing/storage infrastructure for VERDA

Within VERDA, we established a dedicated DataHUB for the TRR356, following the concepts outlined above. Thus, a tailored RDM solution is provided with appropriate availability, customized access control and flexible configuration, with, e.g., the GitLab LFS feature lifting limits on the sizes of datasets. The DataHUB is complemented by dedicated storage and high-performance and cloud computing infrastructure of the university computing centres (ZDV, LRZ). Data transfers are enabled via Git commands addressing the DataHUB and via the established mechanisms of the centres. To meet the specific needs of the PlantMicrobe projects, VERDA additionally integrates tools such as ELabFTW, OMERO, Matrix and JupyterHub. Together, these platforms enable structured documentation of complex experiments, standardized management and sharing of data, interactive analyses of plant-microbe interactions, and collaborative exchange on ongoing research. While computing and write access to VERDA are restricted to TRR356 members and collaborators, the public repositories within the DataHUB are accessible via the web. Reference setup recipes for VERDA components will be shared with the community, particularly with projects adopting DataPLANT principles.

## How does VERDA relate to institutional and international data repositories?

VERDA, as a FAIR project data platform, does not replace data sharing and publication. Rather, it is designed to assist researchers in organizing metadata alongside their data for submission to repositories like those run by the EMBL-European Bioinformatics Institute (EMBL-EBI), the National Center for Biotechnology Information (NCBI), or the DNA Data Bank of Japan (DDBJ), which have long supported biological research [12]. For various TRR356 subprojects, submission to these repositories is practically mandatory due to publisher requirements and best practice standards. While these repositories offer standardized data publication processes and varying levels of support for metadata submission, annotating data is often demanding, and the resulting metadata frequently lacks completeness. While minimal metadata standards are required, data re-users frequently need to consult original publications to fully understand materials, systems, and methods. By integrating annotation early in the data collection process through ARCs in VERDA, we aim to improve metadata quality, enhancing both reusability and machine readability for big data analysis. NFDI4Microbiota and DataPLANT have created templates providing ontology annotations to support data submission to international repositories. NFDI4Microbiota offers metadata standards for different sample types, while DataPLANT’s metadata templates align with the minimum requirements of major repositories.

## Why should researchers care about FAIR principles and dataset publication?

Public research funding agencies increasingly require adherence to FAIR and open data principles to promote reproducibility as a core scientific principle. VERDA supports our plant microbe researchers in meeting these requirements while offering direct benefits. The RDM and data analysis framework of DataPLANT and VERDA improves data organization within individual labs and streamlines data exchange across groups. While publishing papers and generating novel ideas remain central to scientific success, publishing datasets, making them citable via PIDs, provides additional opportunities for academic recognition, especially for datasets not featured in traditional publications. Linking data publication in an ARC to a research article provides a time-stamped public record, offering a measure of protection against being scooped [13]. This is particularly valuable in emerging research areas like microbiome-driven plant trait manipulation, where rapid data exchange can accelerate hypothesis testing and cross-consortium collaboration while ensuring credit to the data author is given. As more biological data becomes openly available through international repositories or publications’ supplementary materials, concerns persist about data publications being cited instead of the corresponding research articles. While this issue isn’t fully resolved, Wood-Charlson and colleagues [14] suggest that researchers can treat data publications as an asset, rather than a threat. In this spirit, we are confident that VERDA will highlight the value of well-designed RDM and FAIR data practices in advancing scientific research.
